# The Book World of Medicine and Science

**Published:** 1905-08-12

**Authors:** 


### The Book World of Medicine and Science.

Studies in General Physiology. By Jacques Loeb, Professor of Physiology in the University of California. (Chicago: The University of Chicago Press. London: T. Fisher Unwin. 1905. Two vols. Price £1 11s. 6d.)

This contribution to the *Decennial Publications of the University of Chicago* is a collection of studies on very diverse subjects. In spite of this diversity, however, a single leading idea permeates all the studies—namely, "that it is possible to get the life phenomena under our control, and that such control, and nothing else, is the aim of biology." In view of work recently done in this country this collection of some of Loeb's most important experiments and results will be of great value and interest to all scientific workers, especially in biology, physiology, and psychology. It is impossible to give a short *resumé* of these valuable papers collectively. Part I. comprises 15 studies, beginning with the heliotropism of animals and its identity with the heliotropism of plants, and ending with the physiological effects of lack of oxygen. This part also contains an elaborate paper—Contributions to the Brain Physiology of Worms. The first paper in Part II. deals with the Influence of Light on the Development of Organs in Animals, the last is written on the Methods and Sources of Error in the Experiments on Artificial Parthenogenesis. In the chapter on Instinct and Will in Animals the author states emphatically that his investigations on the heliotropism of animals have proved that what has been taken for the effect of "will" or "instinct" is in reality the effect of light, of friction, of chemical and other physical forces. He shows by experiment that the orientation of some animals (Spirographis Spallanzanii) is determined by the direction of the rays of light, in other cases the so-called "instinct and free will" is really some definite and controllable cause, *e.g.* gravity, contact with solid bodies, chemical forces, etc. The careful observer will sooner or later find some cause or combination of causes to account for the "instinct or will" of the animal in regard to its movements, and having found such cause he can without fail determine the orientation. In this connection it must not be forgotten that life-phenomena are not dependent solely on the external causes acting on the organism; the conditions present within the organism have to be reckoned with and these are variable. Summarising the results of his well-known experiments on the physiological effects of ions, Loeb states that the addition of small amounts of a very dilute acid or alkali brings about a great increase in weight (absorption of water) in an isolated muscle immersed in physiological salt solution. With the inorganic acids, such as nitric, hydrochloric, sulphuric, this increase in weight is solely a function of the number of hydrogen ions contained in the unit volume of the physiological salt solution. With the bases lithium, sodium, potassium, strontium, and barium hydrate, this increase in weight is solely a function of the number of hydroxyl ions contained in the unit of volume of the physiological salt solution. The substance of an address delivered in 1897 on the Physiological Problems of To-day is given in paper 21. This is well worth the careful consideration and thought of all lovers of physiology.

By the publication of these two volumes the University of Chicago has conferred a boon on biologists of all lands. Each student will find his scientific imagination stimulated and his horizon widened by reading Professor Loeb's studies in general physiology.

The Child's Diet. By T. Sadler Curgenven, M.R.C.S. (London: H. K. Lewis, 1905. Pp. 96. Price 1s. 6d.)

The dietary of childhood has been so much written about within recent years, that further literature on this subject ought to have at least the stamp of freshness and originality. We do not find either in this book. The author believes that less starch and sugar should enter into the dietary of children, and probably most medical men will agree with him.

Electrical Methods in the Treatment of Affections of the Stomach and Intestines. By G. Herchell, M.D. (London: A. Siegle, 1905. Pp. 110. Price 3s. 6d.)

The author of these papers gives a concise and clear account of the methods of application of the various forms of electrical current now used in the treatment of disease. The rapid development of electro-therapeutics has not been accompanied by a corresponding enlightenment of the many as to the action and uses of electrical currents upon the tissues and organs of the body. With the discovery of the newer forms of electricity their properties have to be discovered and tried. The mode of applying electrical current is very important, and as this is the principal difficulty with most people the author's practical hints are of considerable value. Several cases are quoted at the end of the book which the author considers to have benefited by the application of electricity. The only fault to find is that these cases seem to have suffered from symptoms which might well be relieved by the dieting, etc., which was carried out in conjunction with electrical treatment. This the author fairly admits. His cases would be of greater value if the probable cause of the various gastric symptoms had been elucidated. In the first part of the book there are given some reasons for the use of electrical treatment, but the effects observed may be due to either direct or indirect electrical stimulation. There is great need for the study of the effects of electricity on the glands, nerves, and organs of the body independently of disease. Such arguments as the author puts forward do not tend to convince one of the efficacy of electrical treatment.

A System of Clinical Medicine. By Thomas D. Savill, M.D., Lond. Vol. II. (London: J. and A. Churchill. 1905. Pp. 445.)

It is now some time since the first volume of Savill's "System of Clinical Medicine" was published, and those who are acquainted with it may feel assured that the high standard of Vol. I. has been maintained in Vol. II. recently published. The same plan of dealing with diseases is followed; the same care and attention has been given to make the work useful and interesting. The book, which is essentially one for the general practitioner, comprises chapters on General Debility, Pallor and Emaciation, Symptoms Referable to the Extremities, Symptoms Referable to the Skin, Symptoms Referable to the Nervous System, and an Epitome of Clinical Bacteriology and the Examination of Pathological Fluids. The two volumes are separately indexed and are more or less independent of each other. Dr. Savill is to be congratulated on the systematic and practical way in which he has dealt with clinical medicine.

